# Adapting crowdsourced clinical cancer curation in CIViC to the ClinGen minimum variant level data community‐driven standards

**DOI:** 10.1002/humu.23651

**Published:** 2018-10-11

**Authors:** Arpad M. Danos, Deborah I. Ritter, Alex H. Wagner, Kilannin Krysiak, Dmitriy Sonkin, Christine Micheel, Matthew McCoy, Shruti Rao, Gordana Raca, Simina M. Boca, Angshumoy Roy, Erica K. Barnell, Joshua F. McMichael, Susanna Kiwala, Adam C. Coffman, Lynzey Kujan, Shashikant Kulkarni, Malachi Griffith, Subha Madhavan, Obi L. Griffith, Clinical Genome Resource Somatic Working Group and Clinical Interpretation of Variants in Cancer team members

**Affiliations:** ^1^ McDonnell Genome Institute Washington University School of Medicine Saint Louis Missouri; ^2^ Baylor College of Medicine Houston Texas; ^3^ Biometric Research Program, Division of Cancer Treatment and Diagnosis National Cancer Institute Rockville Maryland; ^4^ Vanderbilt‐Ingram Cancer Center Nashville Tennessee; ^5^ Georgetown Lombardi Comprehensive Cancer Center Washington District of Columbia; ^6^ Keck School of Medicine University of Southern California Los Angeles California; ^7^ Baylor Genetics Houston Texas; ^8^ Dan L. Duncan Cancer Center Houston Texas

**Keywords:** cancer, CIViC, ClinGen, ClinVar, curation

## Abstract

Harmonization of cancer variant representation, efficient communication, and free distribution of clinical variant‐associated knowledge are central problems that arise with increased usage of clinical next‐generation sequencing. The Clinical Genome Resource (ClinGen) Somatic Working Group (WG) developed a minimal variant level data (MVLD) representation of cancer variants, and has an ongoing collaboration with Clinical Interpretations of Variants in Cancer (CIViC), an open‐source platform supporting crowdsourced and expert‐moderated cancer variant curation. Harmonization between MVLD and CIViC variant formats was assessed by formal field‐by‐field analysis. Adjustments to the CIViC format were made to harmonize with MVLD and support ClinGen Somatic WG curation activities, including four new features in CIViC: (1) introduction of an assertions feature for clinical variant assessment following the Association of Molecular Pathologists (AMP) guidelines, (2) group‐level curation tracking for organizations, enabling member transparency, and curation effort summaries, (3) introduction of ClinGen Allele Registry IDs to CIViC, and (4) mapping of CIViC assertions into ClinVar submission with automated submissions. A generalizable workflow utilizing MVLD and new CIViC features is outlined for use by ClinGen Somatic WG task teams for curation and submission to ClinVar, and provides a model for promoting harmonization of cancer variant representation and efficient distribution of this information.

## 1 INTRODUCTION

Whole genome sequencing of the first cancer genome and subsequent efforts to survey the pan‐cancer mutational landscape greatly expanded the potential use of cancer variants for research, drug development, and clinical applications (Hudson et al., 2010; Ley et al., 2008; Weinstein et al., 2013). Clinical application of Next Generation Sequencing (NGS) has enhanced molecular profiling capacity (Kamps et al., 2017). NGS sequencing methods are now commonly used in personalized clinical cancer care (Chang et al., 2017; Green et al., 2016). However, NGS also yields increasing numbers of variants that predominantly are of unknown significance and compounds the challenge of variant interpretation (Good, Ainscough, McMichael, Su, & Griffith, 2014; Kamps et al., 2017). As clinical analysis of large volumes of patient variant data becomes increasingly difficult, inconsistencies increase both in variant interpretation and reporting between laboratories (Harrison et al., 2017). This issue is compounded by propagation of these inconsistencies to widely accessed knowledgebases (Hoskinson, Dubuc, & Mason‐Suares, 2017; Yorczyk, Robinson, & Ross, 2015). This underscores the need for regularized clinical classification and representation, as well as open distribution of standardized somatic cancer variant knowledge (Amendola et al., 2015; Shah & Nathanson, 2017).

In order to create consistency and transparency in somatic variant interpretation, the Association of Molecular Pathology (AMP) has recently published a set of guidelines for somatic variant interpretation in cancer, which is seeing steady adoption across multiple platforms (Li et al., 2017). However, currently the field of somatic cancer variant classification is still in development, especially when compared to variant interpretation for germline or Mendelian disorders (Richards et al., 2008; Richards et al., 2015). Besides the AMP cancer variant interpretation guidelines, there have been several other proposed systems for somatic cancer variant classification, which focus on variant therapeutic value (actionability), broader clinical value, or use more complex bioinformatic approaches to the problem (Hoskinson et al., 2017; Sukhai et al., 2016; Van Allen et al., 2014). Minimum variant level data (MVLD; described below and in reference) was developed by The Clinical Genome Resource (ClinGen) Somatic WG (WG) to provide a consensus‐based, lightweight, and modular format to transfer somatic variant data of clinical relevance (Ritter et al., 2016). ClinGen is a global National Institutes of Health (NIH)‐funded effort to standardize gene and variant curation, for clinically relevant genetic information, aiding in rapid communication of this information between multiple end users including clinicians, research scientists, and the public. ClinGen works closely with ClinVar, a database of clinically relevant germline and somatic variants, to implement best‐practices in variant curation and presentation (Landrum et al., 2016a). The Somatic Working Group (WG) is in the Clinical Domain of ClinGen, and is composed of over 50 academic and industry stakeholders in cancer research.

Following development of MVLD to harmonize cancer variant somatic data, the Somatic WG has focused on curation efforts described below using the Clinical Interpretations of Variants in Cancer web resource (CIViC—http://www.civicdb.org) as a curation platform (Griffith et al., 2017). CIViC is a free, fully open access knowledgebase and curation interface for cancer variants that may potentially impact the clinical evaluation of a cancer patient. The knowledgebase uses a crowdsourcing approach combined with expert curators from organizations such as ClinGen (Expert Panels) and CIViC‐trained editors to maintain and expand a resource for clinical interpretation of variants. This addresses a critical need by assisting genome scientists in evaluating the large volume of relevant variant data produced by contemporary tumor NGS analysis (Good et al., 2014). CIViC is a knowledgebase, which is currently NIH‐funded, and provides data with no license restrictions or costs to contribute, use, or view.

**Figure 1:**
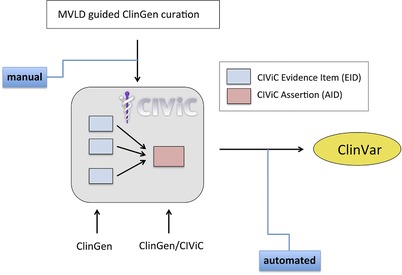
Workflow for Clinical Genome Resource (ClinGen) minimum variant level data (MVLD) formatted somatic cancer variant submission to ClinVar utilizing the Clinical Interpretations of Variants in Cancer (CIViC) assertion. MVLD formatted variant data is used by ClinGen curators to manually generate evidence items using CIViC interface. ClinGen and CIViC curators and editors collaboratively use this evidence to generate CIViC assertions. Assertions are submitted to ClinVar via an automated program

This work reports on a collaborative effort between the ClinGen Somatic WG and CIViC team to employ MVLD and new features developed in the CIViC database for cross‐platform curation of somatic cancer variants and downstream automated submission to ClinVar (Figure 1). Here we describe harmonizing the CIViC somatic variant representation with that of MVLD, and offer a curation workflow for somatic cancer variants that aligns the MVLD representation with the CIViC somatic assertion format. Further, we have automated the transformation of CIViC somatic assertions into ClinVar submissions for consumption by the broader biomedical research community, and provide the code, via GitHub, that enables this transformation to the broader community as well. Our ultimate goal is to use data elements developed through working with curation structures like MVLD and platforms like CIViC to inform the streamlining and standardization of cancer curation data in electronic medical records (EMR), combined with other efforts in this area, such as HL7 Fast Healthcare Interoperability Resource (FHIR) and the GA4GH Genomic Knowledge Standards (GKS) Variant Annotation Task Team (Khalilia et al., 2015; Lawler et al., 2015).

## 2 MATERIALS AND METHODS

### 2.1 MVLD brief description

**Figure 2:**
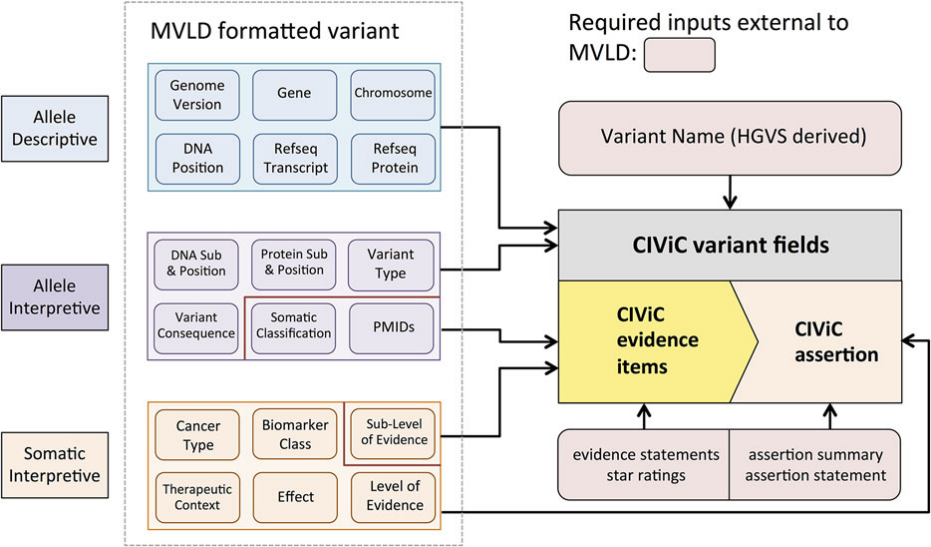
Overview of the relation of minimum variant level data (MVLD) structure to CIViC. The majority of MVLD Allele Descriptive and Interpretive fields map to Clinical Interpretations of Variants in Cancer (CIViC) variant fields, whereas MVLD Somatic Interpretive fields are all associated with CIViC evidence fields, which contain clinical interpretive information for the variant

Briefly, MVLD is a metadata structure that guides selection of ontologies and terminologies (Ritter et al., 2016). MVLD organizes data elements into three categories: Allele Descriptive, Allele Interpretive, and Somatic Interpretive (Figure 2). The Allele Descriptive fields describe the genomic identifiers of a variant: genome build, gene name, chromosome, DNA position, and RefSeq transcript and protein. The Allele Interpretive fields contain data that helps to understand the likely effect and associated relevant literature identifiers (e.g., PubMed IDs). The Somatic Interpretive fields hold data that pertain to the somatic and clinical relevance of a variant. These fields are as follows: Cancer Type, Biomarker Class, Therapeutic Context, Effect, Level of Evidence, and Sub‐Level of Evidence. For a somatic variant, the Level of Evidence captures the interpretation framework used for variant assessment and is conceptually similar to the “assertion criteria” in ClinVar. Although initially published with an example in the Level of Evidence field from the Cancer Driver Log (CanDL), the MVLD has been updated and adopted the interpretive tiers from the AMP guidelines (Damodaran et al., 2015; Li et al., 2017). It is important to note that many somatic variant interpretive schemata could be recorded in the Level of Evidence field (Parsons et al., 2016). Additionally, at the current time, MVLD is tailored for somatic single nucleotide variants (SNVs) and small insertion and deletion (indel) variants, with the intention to expand for relevant somatic events, such as RNA fusions, gene amplifications, and chromosomal rearrangements.

### 2.2 Curating MVLD formatted variants in CIViC: A workflow method

The CIViC interface is used for variant curation and the creation of variant assertions. The interface enables not only submission of content, but also editing, approval, and discussion regarding changes between curators and editors. Furthermore, it provides tracking and recording of all of these actions, allowing transparency of CIViC curations. In this proposed workflow, the CIViC interface is used to both accept evidence entries from MVLD‐formatted and precurated data using general CIViC moderation protocols and to subsequently create variant assertions. An assertion in CIViC is a curation structure built from evidence items (EIDs; structured clinical data extracted from published articles) for a single variant. When the collection of evidence reflects the state of the field, the Association of Molecular Pathologists (AMP) somatic variant interpretation is applied with appropriate Tier and Level.

Although CIViC admits a broad range of gene‐centered variant types, including “bucket” variants such as any mutations within a specific gene or domain, MVLD curation intended for CIViC will focus on SNV and small indel variants. Implementing MVLD with CIViC is best accomplished by a workflow and user optimization, and is not yet scoped for automated transformation of data, although we may yet develop it further. Specifically, MVLD will function as a record of precuration for the Somatic Assertion feature in CIViC in the following workflow: (1) the Somatic WG biocuration team members will curate variants in MVLD format and pull associated PubMed identifiers (PMIDs) into an MVLD record, (2) the MVLD record can then be reassigned to curation team members to pull the PMIDs, review the articles in‐depth, extract CIViC EIDs, and enter them into the interface, (3) upon completion of a series of EID entries, a CIViC Somatic Assertion can be created, and (4) the Somatic WG will review and approve a “final” assertion in CIViC.

### 2.3 Harmonizing MVLD and CIViC: A field‐to‐field analysis

Although the workflow for MVLD‐guided ClinGen curation into CIViC (Figure 2) does not involve an automated mapping of MVLD‐formatted somatic variant data, a field‐by‐field mapping analysis from MVLD into CIViC was performed to gauge harmonization of the variant representations (Supporting Information Figure S1a–c). In fields where a natural mapping from MVLD to CIViC was not apparent, workarounds were formalized while maintaining the intent of the respective fields from each system. In cases where no workaround of this nature was apparent, the discrepancy was noted and evaluated, and if deemed important, changes to the CIViC variant format were suggested and implemented. Fields in CIViC that were outside the scope of MVLD were also noted, and assessed for their relevance toward variant harmonization between the two representation formats.

### 2.4 Automated CIViC to ClinVar mapping for submission

A formal mapping based on fields drawn from the assertion and variant subsections of CIViC was constructed (Supporting Information Figure S2a–c), which describes the relationship between CIViC elements and the fields required for variant submission to ClinVar. An object‐oriented python package and command‐line application was developed to query the CIViC database for assertions over the RESTful API, then evaluate and assemble the relevant information for a ClinVar submission from the resultant JSON responses. The CIViC assertion is then used to create the required fields for a ClinVar record. Each record is written to a ClinVar‐compliant submission form for entry into the ClinVar system. This open‐source application is hosted on GitHub at https://github.com/griffithlab/civic2clinvar, under the permissive MIT software license. The CIViC database model will be updated with a feature to track and version ClinVar submissions.

## 3 RESULTS

### 3.1 CIViC development to support ClinGen somatic curation

#### 3.1.1 Somatic Assertions: AMP Tiers and levels

**Figure 3:**
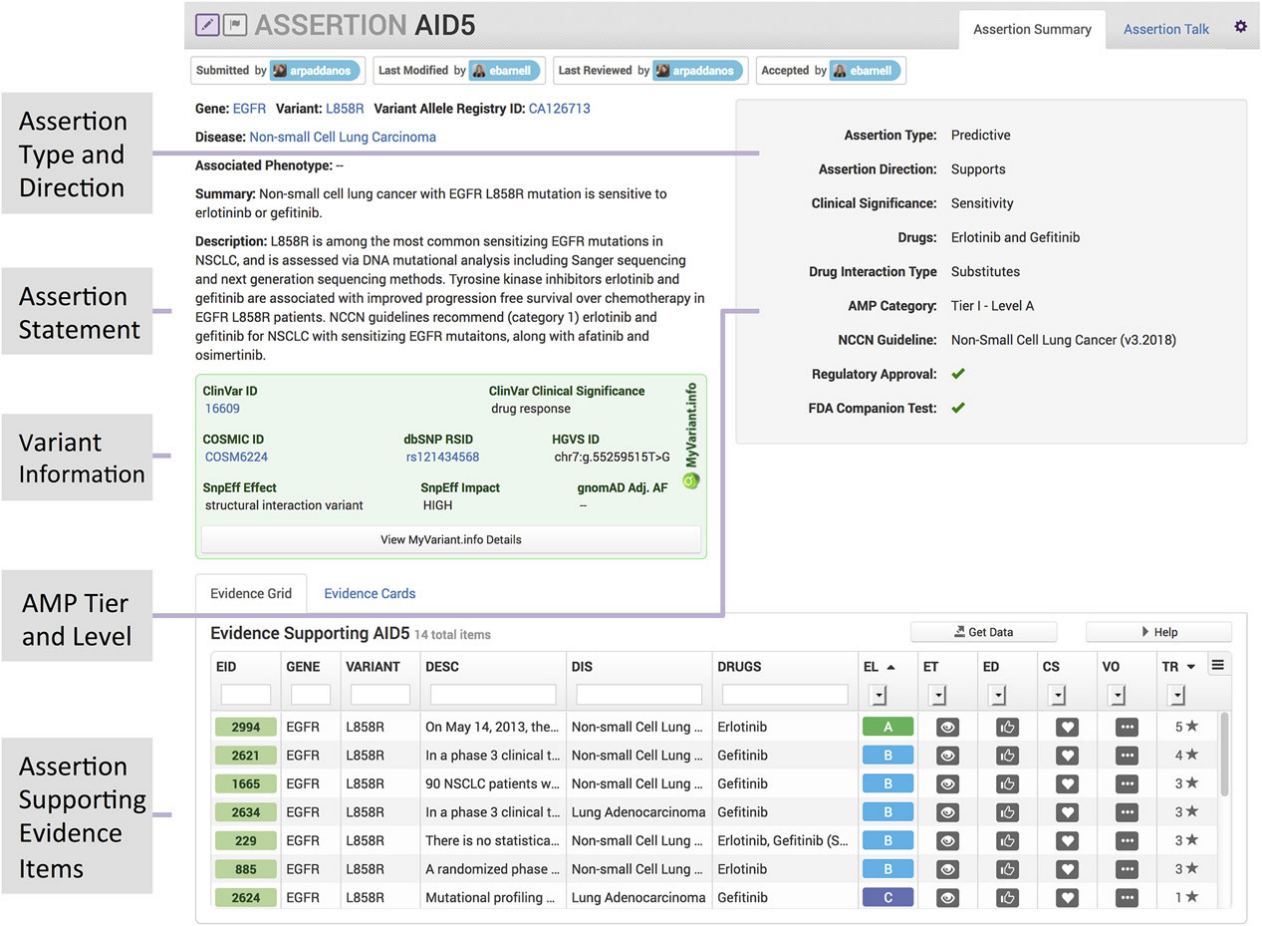
The Clinical Interpretations of Variants in Cancer (CIViC) assertion feature. The new assertion feature collects multiple evidence items and summarizes them into an assertion statement. Variant details are given as well as a list of the evidence items used to construct the assertion with Association of Molecular Pathologists (AMP) Tier and Level

MVLD formatted variants contain AMP somatic variant interpretation guidelines as one of their central fields in the “Level of Evidence” element (Li et al., 2017; Ritter et al., 2016). AMP somatic variant interpretation guidelines assign a Tier and Level to classify a somatic variant. The AMP Tier and Level comprise a state of the field evaluation and take into account all clinically relevant knowledge about a somatic cancer variant in a given disease context. In contrast, the CIViC EID is a granular unit of predictive, diagnostic, prognostic, or predisposing evidence drawn from the literature. In order to support a summary statement about the clinically relevant knowledge for a given cancer variant in CIViC, the Assertion feature was created. To support a new first‐class entity with complete functionality (e.g., commenting, editing), changes to the underlying CIViC database and user interface were made. Novel connections and tables were added to the database schema to support Assertions with API endpoints to support multiple web interface features. New advanced search parameters, navigation pages, help documents, and curation forms were added to the user interface in addition to the complex EID searching and linking required to generate an evidence‐supported assertion. When the number and quality of CIViC EIDs of a certain CIViC somatic Evidence Type (predictive, prognostic, or diagnostic) is deemed sufficient to reflect the state of knowledge in the literature, then a curator may write an Assertion (Figure 3), which summarizes the state of the field. Currently, the sufficiency of evidence for an assertion is determined manually by assessing the literature and EIDs; however, as more assertions are created, analysis on contributing factors will help to automate and create standard operating procedures for identification of assertion‐ready variants. Assertions require the curator to apply an appropriate AMP Tier and Level, which in CIViC, range from Tier I Level A to Tier II Level D. In CIViC, such an Assertion clearly links back to the data upon which the Assertion is based, allowing for rapid integration and interpretation in the event of newly published results or the discovery of previously erroneously omitted data.

#### 3.1.2 Organizations feature tracks curation progress and ClinVar attributions at a group level

As CIViC has engaged in more collaborations at the organizational level, a feature to group users into organizations was introduced into the interface (Figure 4a). Every registered CIViC member may belong to one organization, or have no organizational affiliation. An organization page is provided, which features an organization description and list of members (Figure 4b), along with organizational statistics detailing multiple types of curation activity totals and a list of specific curation actions performed by the organization, as well as a list of all EIDs submitted by organization members (Figure 4c). An organization for ClinGen Somatic WG members was made in CIViC (Figure 4a–c), providing proper attribution for this group's efforts throughout the interface and annotating the contributed records for submission to ClinVar using the automated submission process described below. All Somatic Assertions in CIViC will be submitted to ClinVar, and those that have been reviewed by the Somatic WG task teams will be noted as such in the ClinVar submission.

Figure 4Clinical Interpretations of Variants in Cancer (CIViC) organizations page. The CIViC organizations feature is displayed on the community page of the web interface (https://civicdb.org/community/organizations). (a) Each organization icon serves as a link to an organization's page. (b) The organization's page gives a brief description of the organization and lists CIViC curators and editors that are members. (c) Detailed statistics for the organization are provided as well as a summary of organization CIViC activity. A downloadable list of CIViC evidence items submitted by the organization is also provided

**Figure:**
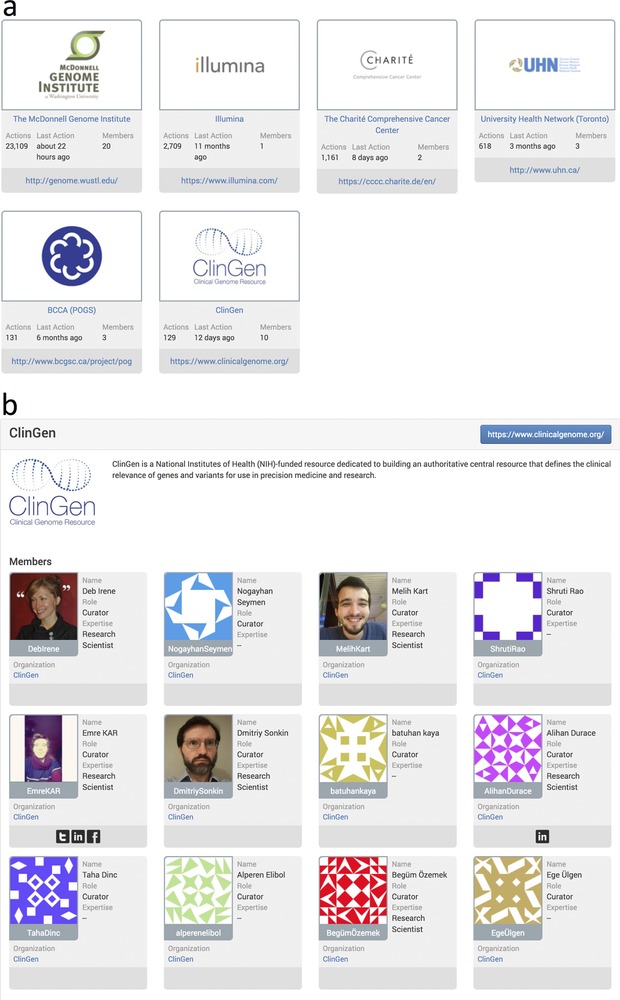

**Figure:**
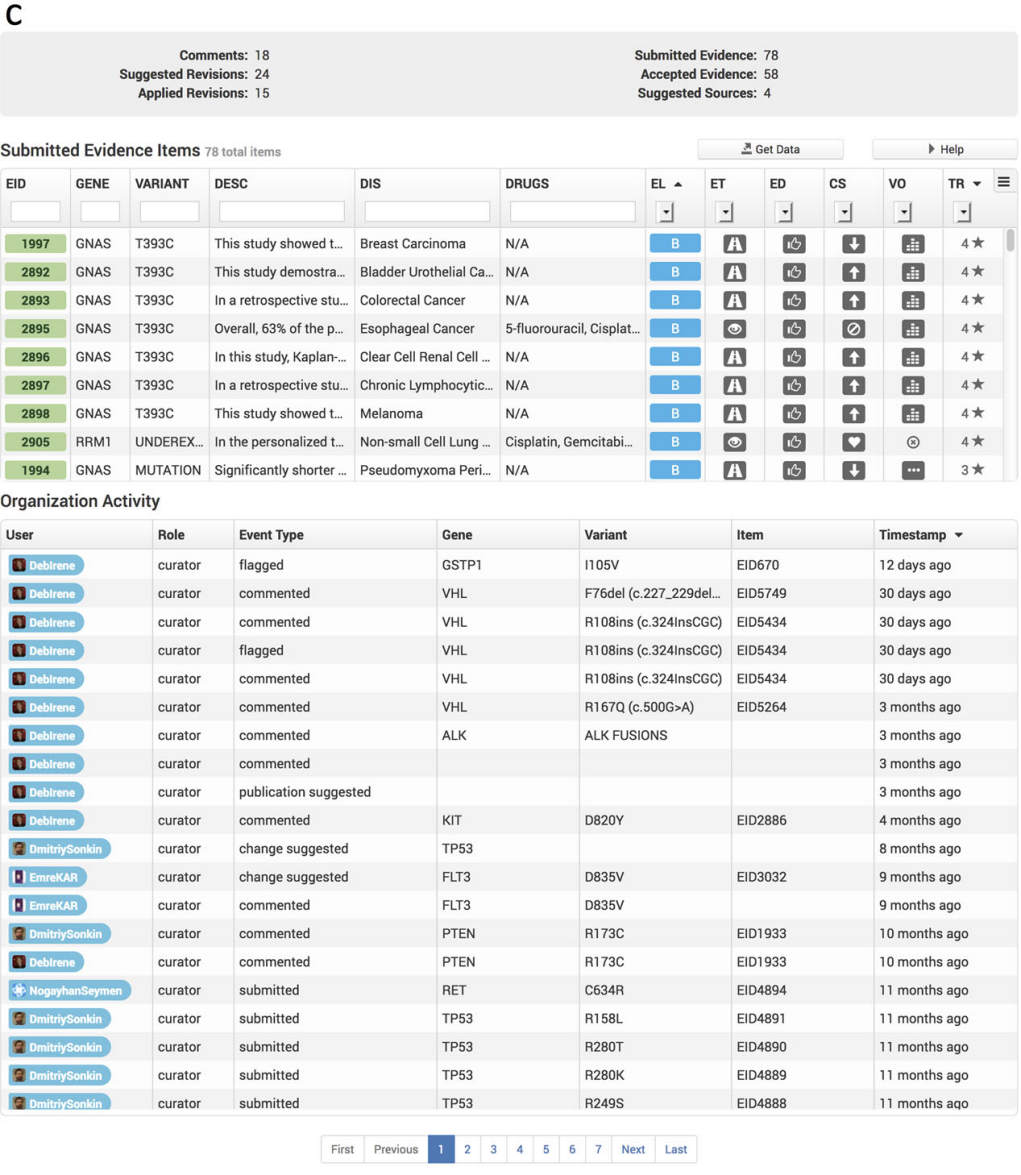

#### 3.1.3 Utilizing ClinGen allele registry in CIViC

**Figure 5:**
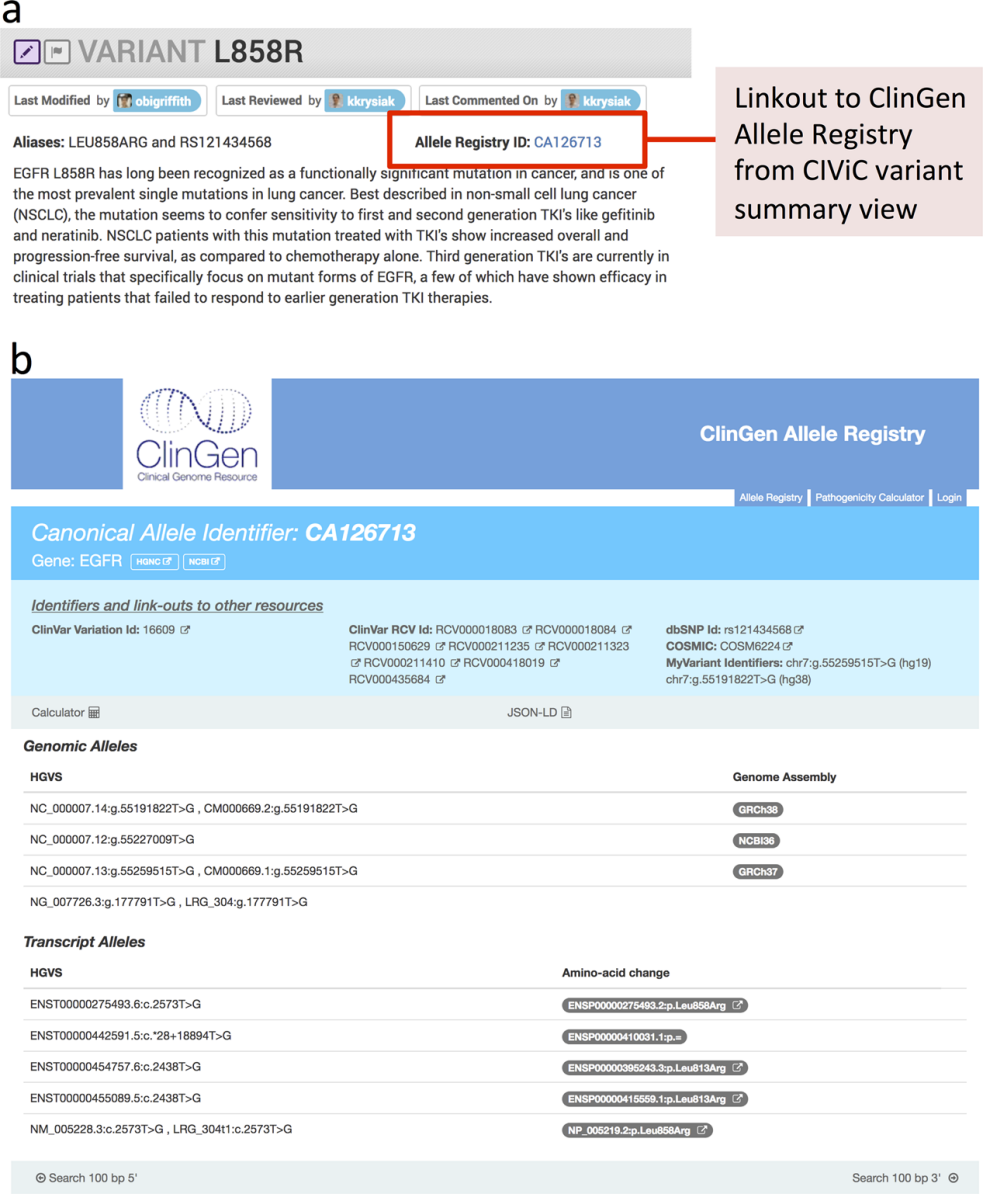
Clinical Genome Resource (ClinGen) Allele Registry in the Clinical Interpretations of Variants in Cancer (CIViC) interface. (a) A link to the ClinGen Allele Registry has been added to the CIViC variant page. For single nucleotide variants (SNVs) and small insertions and deletions, the ClinGen allele is identified automatically once CIViC variant coordinates are curated. (b) ClinGen Allele Registry page for a SNV type variant

ClinGen Allele Registry provides unique and dereferenceable identifiers for every registered variant (https://reg.clinicalgenome.org, and see Patel et al in this issue). In addition, the Allele Registry generates mapping to various genome assemblies and transcripts using Human Genome Variation Society (HGVS) nomenclature. Coordinate descriptions provided with each variant in CIViC are used to find identifiers from the ClinGen Allele Registry (CAIds) using REST‐APIs. Provided identifiers are then used to generate click‐through links at the CIViC variant level and Assertion pages (Figure 5a and 5b). In the future, we will also utilize the registration services to automate the registration of new alleles if the variant of interest is not already present in the Allele Registry.

#### 3.1.4 Mapping CIViC to ClinVar for variant submission

**Figure 6:**
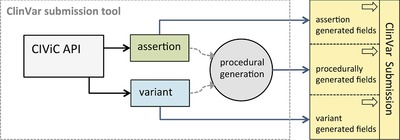
Overview of automated ClinVar submission procedure. The ClinVar submission tool distinguishes three types of fields in the ClinVar submission form: those that accept Clinical Interpretations of Variants in Cancer (CIViC) assertion fields, those that accept CIViC variant fields, and those that require procedural generation to determine the field value

Using fields made available with the addition of the Assertion feature, we have built a formal procedure for mapping CIViC fields into ClinVar submission fields for SNV and indel, along with a python tool for implementing this formalism (Figure 6). This tool is easily expanded to a wider array of variant types. The mapping from CIViC to ClinVar fields is implemented with three types of data: CIViC variant field data, CIViC assertion field data, and procedurally generated entries into the ClinVar sheet. CIViC variant fields map into ClinVar submission fields with no alterations (Supporting Information Figure S2a). CIViC assertion fields map into ClinVar fields with two fields (Condition ID Type and Condition ID Value) requiring some additional logic to properly format the entry (Supporting Information Figure S2b). Finally, there are a set of ClinVar submission fields that require procedural generation based on logic that depends on the CIViC submission fields. These fields along with the logic required for generating them are detailed in the Supporting Information Figure S2c. An example of the output of this procedure using a specific assertion (AID5 from Figure 3) is also shown in the Supporting Information Figure S3).

### 3.2 Harmonizing and relating MVLD to CIViC for streamlined curation

In order to assess harmonization between the MVLD and CIViC‐formatted somatic variant, we performed a field‐by‐field mapping of MVLD into CIViC after completion of the CIViC Somatic Assertions update, and analyzed which fields map from MVLD to CIViC in a natural way, which fields require workarounds to map, and which fields did not admit a workaround for mapping. The latter fields in CIViC were analyzed and suggested changes to CIViC were proposed.

#### 3.2.1 Mappable MVLD to CIViC fields

Because MVLD was implemented as a modular, minimal data structure, and as CIViC and MVLD have an ongoing collaboration, CIViC has already adopted some standards that are suggested in MVLD and in common use by many variant curation databases, such as the use of HGVS nomenclature. A review of the MVLD fields shows that all six MVLD allele descriptive fields map cleanly into CIViC (Supporting Information Figure S1a). From the MVLD allele interpretive fields, DNA Sub and Position, Protein Sub and Position, Variant Consequence, and PMIDs have close analogs in CIViC (Supporting Information Figure S1b). Among the MVLD somatic interpretive fields, all fields map except for Biomarker Class and the expert opinion Sub‐Level of Evidence (Supporting Information Figure S1c), which are discussed below.

#### 3.2.2 Relatable MVLD to CIViC fields

Some fields do not map from MVLD to CIViC in a direct fashion, but admit a relation or adaption to the mapping that does not require changes to the variant format of either MVLD or CIViC. One such field is the MVLD conception of Somatic Classification, which requires that a variant be confirmed somatic or germline, which requires matched tumor and normal control sequencing for confirmation. CIViC does not require matched normal for cases where there is a strong reason to make the assumption that the variant is somatic, as is the case in many cancer studies that do not perform this verification. In cases where somatic origin is less clear, CIViC uses the term Unknown. To work around this difference, ClinGen SWG curators will provide details on control sequencing in the CIViC Evidence Statement. This level of experimental detail is already often voluntarily employed by CIViC curators. Although the MVLD Variant Type has no direct analog in CIViC, the MVLD Variant Consequence naturally maps to the CIViC Variant Type field, which is drawn from the Sequence Ontology (Eilbeck et al., 2005). Another MVLD field that does not map into CIViC is the Expert Opinion Sub‐Level of Evidence (Supporting Information Figure S1c). As CIViC relies exclusively on primary published data documented with a PMID, expert opinion has no analog in the CIViC data model. This is addressed by curation workflow handling of PMIDs, outlined below.

#### 3.2.3 Nonrelatable MVLD to CIViC fields and implemented CIViC modifications

**Figure 7:**
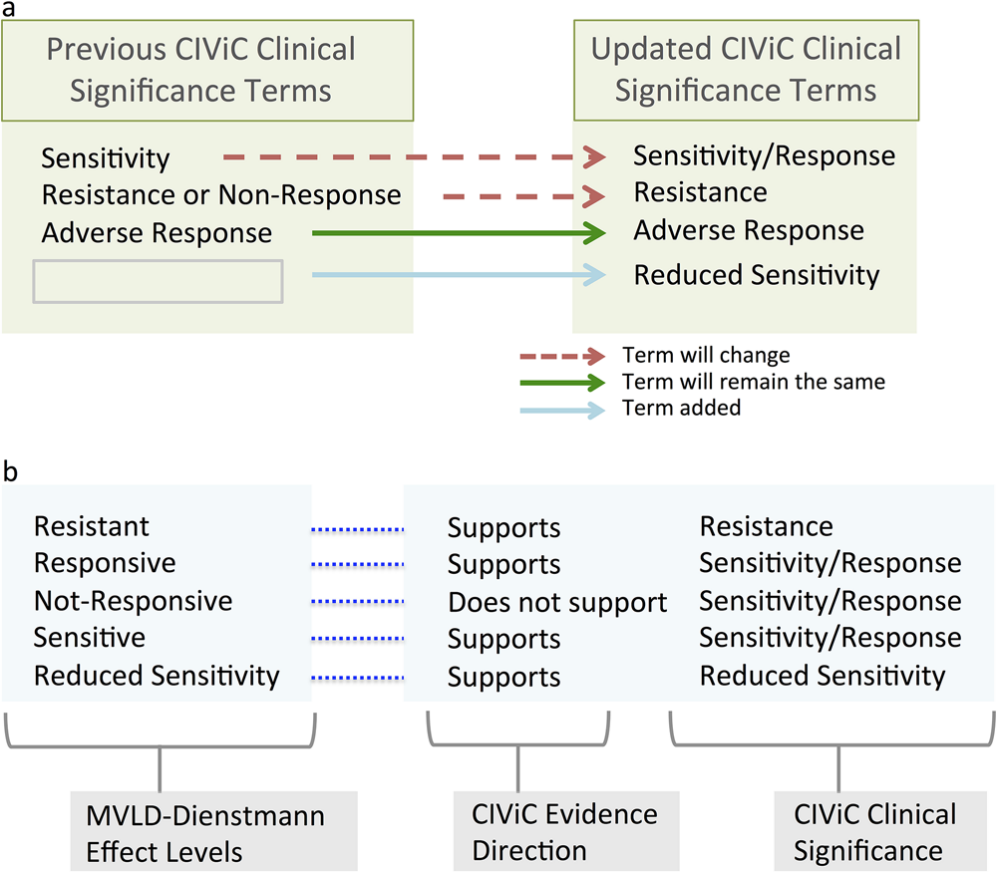
Updating Clinical Interpretations of Variants in Cancer (CIViC) clinical significance terms for harmonization with other standards. (a) Sensitivity is expanded to indicate sensitivity and responsiveness, and resistance or Nonresponse is restricted to Resistance. The new term Reduced Sensitivity adds gradation to Clinical Interpretations of Variants in Cancer (CIViC) evidence. (b) Mapping of the structure of minimum variant level data (MVLD) terms adopted from Dienstmann onto updated CIViC terms

Other fields in MVLD do not admit a mapping into CIViC, and also did not admit a workflow modification to handle this incongruence. One such set of fields are MVLD's Somatic Interpretive Effect fields that are adopted from Dienstmann, and consist of five levels as follows: Resistant, Responsive, Not‐Responsive, Sensitive, and Reduced Sensitivity (Dienstmann et al., 2014). In CIViC, the Effect fields are mainly used for the Predictive biomarker class, as opposed to the Diagnostic and Prognostic classes, whereas in MVLD, the Effect field is optional and may be used for prognostic class. In the CIViC EID and Somatic Assertion, data comparable to the MVLD Effect field are contained by two metadata fields—the Evidence Direction and Clinical Significance—that are paired to the Evidence Type (MVLD Biomarker Class). CIViC's Evidence Direction and Clinical Significance do not cover all of the Effect fields employed by MVLD adopted from Dienstmann et al. (2014). In order to capture these fields, CIViC has implemented changes to the Clinical Significance fields (Figure 7a). The term Sensitivity is changed to Sensitivity/Response, and the term Resistance or Non‐Response is changed to Resistance. Also, the term Reduced Sensitivity is added to the CIViC fields. With these changes in place, a mapping of the five terms adopted from Dienstmann is available in CIViC (Figure 7b), with the exception that the Dienstmann terms Sensitive and Responsive have been reduced to the single compound term Sensitivity/Response in CIViC. We note that in all cases, further nuances to categories can be added to text in the Evidence Statement.

#### 3.2.4 Comparison of MVLD and CIViC handling of PubMed IDs

Although in an MVLD representation of a somatic variant, the PMID fields are optional to allow for unpublished case data, it is recommended and required in the proposed curation workflow that the PMIDs constitute support for the AMP somatic variant interpretation assigned to the MVLD variant. The collection of PMIDs in an MVLD record forms the evidence sufficient to support the AMP somatic variant interpretation. In contrast, CIViC relies upon individuated EIDs for a variant. It is not required in CIViC that a variant's collection of EIDs is representative of the field. Instead, when the quantity and quality of EIDs in CIViC reach the point of summarizing the state of the field for a given variant and disease, then an assertion can be written for this particular combination of variant and disease.

#### 3.2.5 Required CIViC fields

Curation of diagnostic and prognostic evidence in CIViC requires an evidence direction and Evidence Statement, whereas in MVLD, these fields are left as open text. This is solved via a guideline to MVLD precuration, which requires curators to assign evidence direction when dealing with diagnostic or prognostic MVLD Biomarker Classes. CIViC also employs a star rating system for submitted evidence, which is a rating of the quality of a unit of evidence submitted to CIViC—in the form of an EID—which is drawn from a publication. ClinGen curators who have read and assessed the evidence being submitted assign these ratings upon submission in the CIViC interface.

### 3.3 Variant curation through somatic expert review and ClinVar submissions

#### 3.3.1 Variant curation standard operating procedure and task teams

The Somatic WG has adopted much of the structure of ClinGen Germline Expert Panels for their curation task teams, and is formalizing the process of Somatic Expert Panels. The Somatic WG is divided into curation task teams focused on cancers and genes, including the following: Pediatric Somatic Cancers, Pancreatic Cancers, Nonsmall Cell Lung Cancers, and Somatic TP53 Mutations. Each task team defines team leaders and participants, a gene and variant set, a monthly meeting agenda, and mission statement that includes curation targets with the available workforce. In an initial round of curation prior to task team formation, the Somatic WG added ∼80 EIDs to CIViC, from a set of ∼30 high‐impact cancer genes that lacked somatic assertions in ClinVar. Following this, the task teams have each established functional curation plans. Here, we review the Pediatric Somatic Working Group (PSWG) curation plan as an example. The PSWG has defined a set of pediatric cancer genes in specific childhood tumor types, and has identified and prioritized variants in disease‐gene pairs using Mastermind, a literature mining search tool (https://mastermind.genomenon.com/). A gene‐disease search in Mastermind produces a list of variants, and these are prioritized based on the following: (1) overall absence of curated data in CIViC, (2) the number of article hits against the Mastermind search, (3) the number of hits against a PubMed search, and (4) review of variants in pediatric‐relevant datasets (Chakravarty et al., 2017; Ma et al., 2018). The variants are discussed and vetted with experts on the WG call. After an initial round of curation, curators assemble the MVLD record from a high‐level literature review and pull in relevant articles. In this step, experts in the WG may be familiar with relevant articles and list them in addition to articles listed in the MVLD record. The MVLD can then be assigned to trainees and onboarding curators to extract EIDs from the PMIDs and assemble into a Somatic Assertion. The Pediatric Task Team reviews the Somatic Assertions on monthly calls and provides feedback on curation and interpretation of the EIDs.

#### 3.3.2 Somatic WG moderation in CIViC

Currently, CIViC editors moderate ClinGen Somatic WG submissions. Moderation requires a curator with editor‐level status to review the literature used to create an EID, after which an editor can directly accept the submission, or if deemed necessary, revise the entry by suggesting revisions. Members of the Somatic WG who specialize in somatic biocuration will receive “editor‐level” status to moderate submissions from the ClinGen Somatic WG.

#### 3.3.3 Somatic WG curation and submission to ClinVar

After ClinVar submission of a small test set of somatic assertions, a larger set of 500 submissions is expected to be completed by end of 2018. As part of an ongoing effort, CIViC will submit all assertions to ClinVar on a biannual basis. As we further develop and solidify the submission process and as the rate of assertions in CIViC increases, we may seek to increase the number of submissions. Assertions generated by ClinGen Somatic WG will use the CIViC organization's functionality to be labeled as such for ClinVar submission.

## 4 DISCUSSION

With the publication of the AMP Somatic Variant Interpretation Guidelines and implementation of the Somatic Assertion (Tier and Level) into CIViC, a close homology was attained in the MVLD and CIViC representations of somatic cancer variants. Granular field mapping revealed many points of practical agreement between CIViC and MVLD data models, requiring relatively minor modifications to CIViC. Combining the efforts of the ClinGen Somatic WG and CIViC somatic variant interpretation models into a practical curation workflow provides a strong basis for reporting, discussing, and curating the most clinically‐relevant somatic variants in a consensus building and flexible structure that will allow for updates as somatic variant guidelines evolve. Ideally, the detailed provenance of this effort will influence upcoming somatic variant guidelines.

The increasing amount of somatic variants produced by clinical sequencing necessitates rapid curation and dissemination. Currently, there are multiple platforms and portals hosting cancer variant data with a clinical focus, including OncoKB, CanDL, My Cancer Genome, The Jackson Laboratories Clinical Knowledgebase, and ClinVar (Chakravarty et al., 2017; Damodaran et al., 2015; Landrum et al., 2016b; Patterson et al., 2016; Swanton, 2012). This speaks to the need for coordinated efforts such as that presented here to define and relate central data elements. We hope to extend the interoperability further to additional curation platforms. It is relevant here that the way the community shares clinically identified variants is also rapidly evolving. Many journals, such as Molecular Case Studies, NPJ Genomic Medicine, Human Genome Variation, and JCO Precision Oncology, are beginning to accept cancer genetics case studies as a new publication format. These provide a vehicle and a rapid mechanism to share molecular analysis of patients or cohorts alongside their clinical phenotypic information. These n‐of‐1 reports are short standardized reports about genomic variation and variability, especially in relation to a disease or drug sensitivity or resistance. However, many journals require submission of variants or sequencing results to public databases in order to promote data sharing. Databases that rely on PMIDs and literature variant curation may not accept relevant cancer cases due to lack of publication evidence. Cancer Genetics will soon implement a new rapid publication model that will highlight interesting cancer cases and associated variants, with the intention that variant data would be submitted to the journal in MVLD format followed by submission and curation in CIViC after PMID assignment. Cancer medicine will greatly benefit from the large scale dissemination of this case‐based knowledge to a wide community. In addition, precision oncology could be substantially improved from the biocuration and systematic reviews communities coming together, given the emphasis of the former on timely knowledge dissemination and the latter on systematic assessment of the literature and the risk of bias. For example, curated databases like CIViC could be considered as one of the inputs to systematic reviews while at the same time always including outputs from systematic reviews (Boca, Panagiotou, Rao, McGarvey, & Madhavan, 2018). The MVLD–CIViC effort outlined here provides a framework to solve these problems, employing MVLD format standardization and CIViC's commitment to ensure no barriers exist for those seeking access to these findings.

Efforts such as the one presented here demonstrate the utility of MVLD as a central structuring principle for variant representation, which can streamline somatic variant curation, and make lateral transfer of variant knowledge more efficient and rapid as a standardized conception for a somatic cancer variant emerges. Such a framework not only allows for standardization but also allows for integration of data generated by different laboratories to enable novel hypothesis generation for precision oncology. Likewise, commitment to an open data model such as that adhered to by CIViC is essential to enabling this process, which in turn serves to minimize redundant effort in tackling the enormous problem of cancer somatic variant curation and interpretation. Efficient distribution of information, including mappings and automations such as those presented here, further enables rapid adoption of new findings to clinical applications such as panels, or drug development, and standardizations enable more efficient integration, with minimal redundancy, of updated variant interpretations into tools such as EMR. Although a data warehouse, such as ClinVar, could store and serve variant curation data, it is abundantly clear that multiple curation input platforms are preferred by biocurators and those contributing curations, and thus establishing shared core elements is essential to the development and design of curation platforms. Curation effort as a whole is obviously crucial to the success of these efforts, and part of this effort may be incentivized in the form of training the next generation of cancer data scientists, as the curator interested in understanding the current state of the field greatly benefits from these activities.

## Supporting information

Supporting Information

Figure S1. Field by field mapping of MVLD into CIViC. (a) MVLD Allele Descriptive fields map into existing CIViC fields with no alteration. (b) MVLD Allele Interpretive fields map into CIViC fields with some conditions on the mapping for Somatic Classification and Variant Type fields. (c) MVLD Somatic Interpretive fields map into CIViC fields with some conditions on the mapping for Biomarker Class fields and the exception of expert opinion Sub Level of Evidence, which has no analogue in the CIViC format.

Supporting Information

Figure S2. Protocol for automated generation of ClinVar submission on a field by field basis. Three types of fields are distinguished by the process of generating ClinVar submissions from CIViC fields. None of the required ClinVar submission fields is impossible to generate using data either directly obtained or derived from CIViC assertion and related fields. (a) CIViC Variant fields map directly into a subset of required for ClinVar submission. (b) CIViC Assertion fields map into a subset of ClinVar submission fields non‐overlapping with the variant fields, and requires some logic to generate Condition ID Type and Condition ID Value fields. (c) A third type of ClinVar submission field has no direct analogue in CIViC fields but is filled using logic based on CIViC fields or free text.

Supporting Information

Supporting Information

Figure S3. CIViC fields transformed to ClinVar format. A sample transformation from fields of a CIViC assertion (AID5) following the protocol for automated ClinVar submission in the civic2clinvar python tool.
