# Supplementary material for: Adapting crowdsourced clinical cancer curation in CIViC to the ClinGen minimum variant level data community‐driven standards
Source: Hum Mutat. 2018 Oct 11;39(11):1721–32. doi: 10.1002/humu.23651 (PMC6282863; doi:10.1002/humu.23651)
Supplement: Supplementary file 1 — Supporting Information [file HUMU-39-1721-s007.pdf]

Fig. S1a

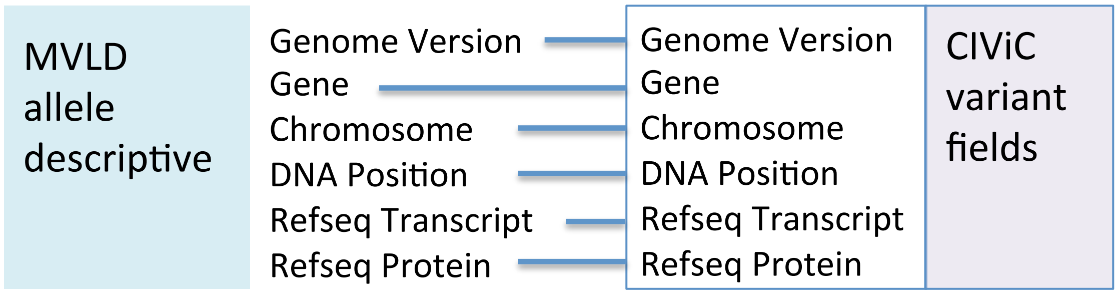

Fig. S1b

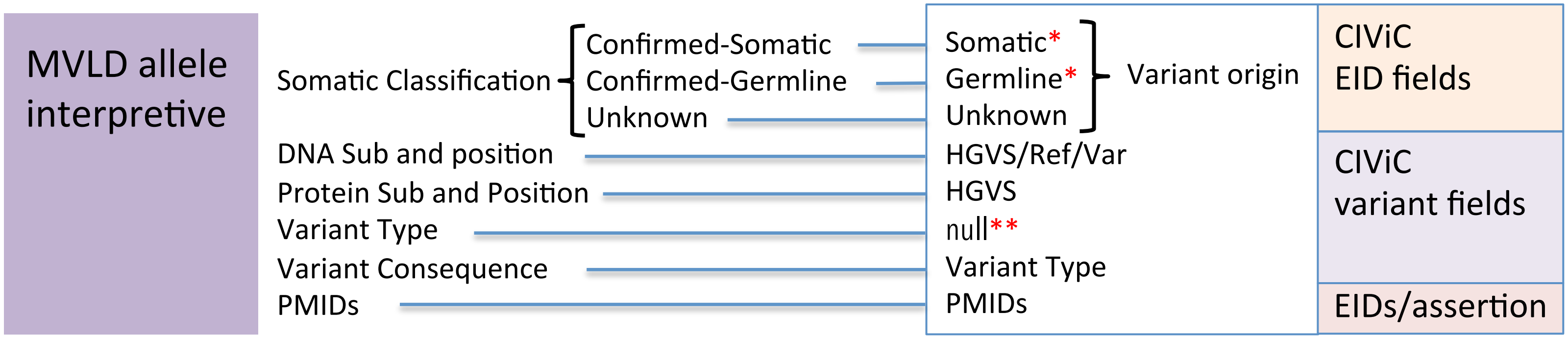

* If MVLD variant is confirmed-somatic, then choose somatic in CIViC EID, and note confirmed somatic in EID evidence statement.

** MVLD Variant Type (SNV, Insertion, Deletion) is inferred from the CIViC Variant Type field, drawn from the Sequence Ontology

Fig. S1c

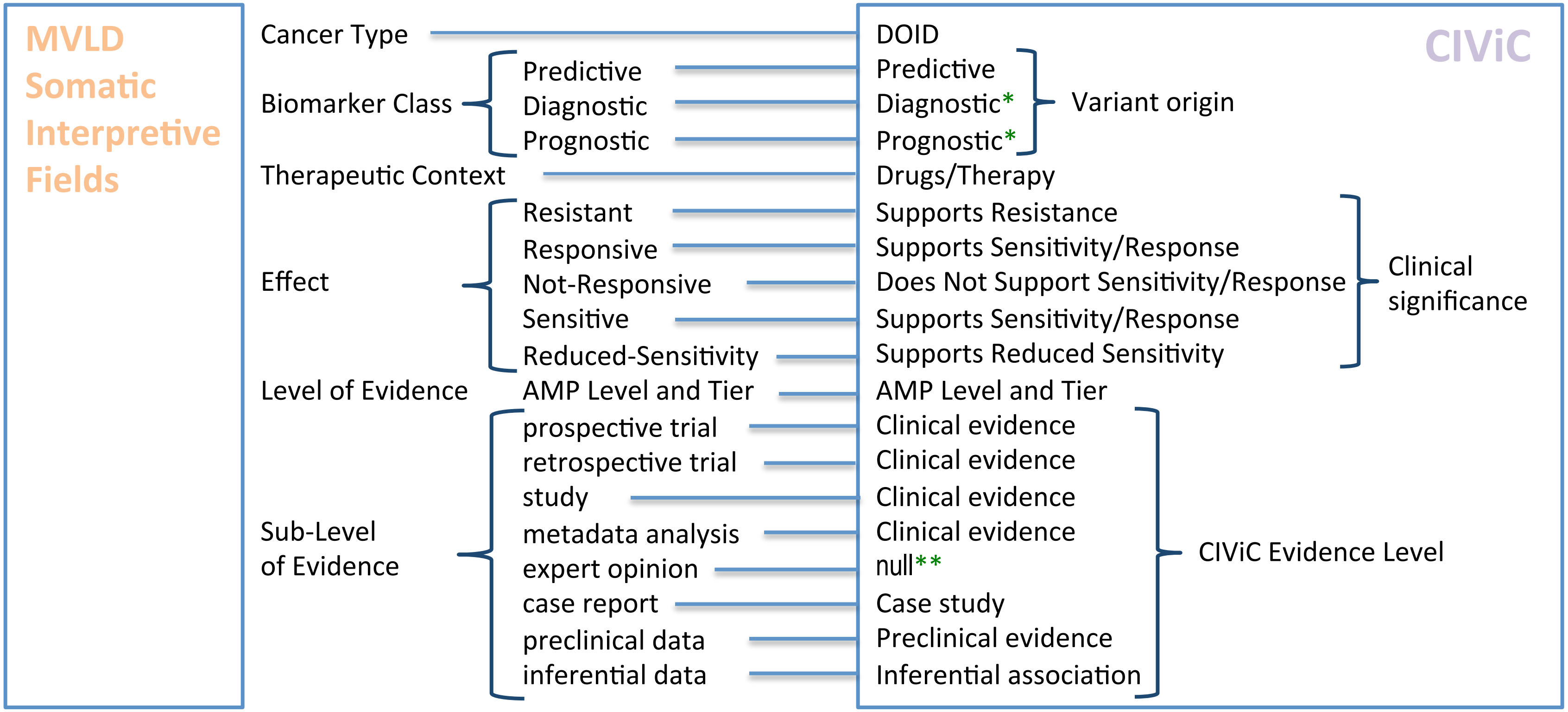

*Diagnostic and Prognostic fields in CIViC require evidence direction, while MVLD is free text.

**MVLD variants based on expert opinion are currently not supported by CIViC, which requires Pubmed ID.

Fig. S2a

Clinvar Field	Column		CIViC Variant Field
Reference sequence	D	←	ensembl transcript
HGVS	E	←	HGVS
Chromosome	F	←	Chr
Start	G	←	Start
Stop	H	←	Stop
Reference allele	I	←	ref
Alternate allele	J	←	var
Variation identifiers	X	←	variant pub data IDs
Alternate designations	Z	←	variant name | Aliases
URL	AB	←	variant URL

Fig. S2b

**Clinvar Field**	**Column**	**CIViC Assertion Field**
##Local ID	A	AID
Gene symbol	C	Gene
Condition ID type	AD	*if assertion has HPO:* HPO
Condition ID value	AE	*if assertion has HPO:* HPO IDs
Preferred condition name	AF	DOID term
Clinical significance citations	AP	PMID list
Citations or URLs for clinical significance without database identifiers	AQ	EID URL list
Comment on clinical significance	AR	Assertion Summary. Statement
Explanation if clinical significance is other or drug response	AS	Direction and Significance
		If assertion is predictive: Drug

Fig. S2c

Clinvar Field	Column	Entry Produced Based on CIViC Fields
Condition comment	AI	optional text giving detail for condition
Clinical significance	AK	*If assertion is predictive:* Drug Response<br>*else if assertion is diagnostic or prognostic:* Other
Collection method	AX	literature only
Allele origin	AY	somatic
Affected status	AZ	unknown
Comment	CO	AMP Tier and Level; comment on CIViC
Private comment	CP	private comment
ClinVarAccession	CQ	Unique Submission Identifier

purple text indicates CIViC field
blue text indicates a string to be entered into ClinVar field
italicized text indicates logic implemented by submission protocol
orange text indicates optional text entered as parameters when running submission protocol
green text indicates unique submission ID

Entry is a direct submission of CIViC field or text
Entry requires logic based on fields from CIViC assertion

Figure S3

ClinVar Field	CIViC data example from AID5
Reference sequence	NM_005228.4
HGVS	NC_000007.13:g.55259515T>G,NM_005228.4:c.2573T>G,ENST00000275493.2:c.2573T>G,NP_005219.2:p.Leu858Arg
Chromosome	7
Start	55259515
Stop	55259515
Reference allele	T
Alternate allele	G
Variation identifiers	dbSNP:rs121434568; COSMIC:COSM6224; ClinVar:16609
Alternate designations	LEU858ARG; RS121434568
URL	https://civicdb.org/events/genes/19/summary/variants/33/summary
##Local ID	AID5
Gene symbol	EGFR
Condition ID type	-
Condition ID value	-
Preferred condition name	non-small cell lung carcinoma (DOID:3908)
Clinical significance citations	PMID:24868098;PMID:18509184;PMID:20038723;PMID:24893891;PMID:15329413;PMID:19147750;PMID:22370314;PMID:24736073;PMID:27102076;PMID:27032107;PMID:21132006;PMID:17877814;PMID:22285168;PMID:24868098
Citations or URLs for clinical significance without database identifiers	https://civicdb.org/links/evidence/276|https://civicdb.org/links/evidence/1665|https://civicdb.org/links/evidence/2621|https://civicdb.org/links/evidence/2625|https://civicdb.org/links/evidence/2624|https://civicdb.org/links/evidence/3811|https://civicdb.org/links/evidence/2634|https://civicdb.org/links/evidence/229|https://civicdb.org/links/evidence/4291|https://civicdb.org/links/evidence/4290|https://civicdb.org/links/evidence/4285|https://civicdb.org/links/evidence/4265|https://civicdb.org/links/evidence/885|https://civicdb.org/links/evidence/2994
Comment on clinical significance	Non-small cell lung cancer with EGFR L858R mutation is sensitive to erlotininb or gefitinib. L858R is among the most common sensitizing EGFR mutations in NSCLC, and is assessed via DNA mutational analysis including Sanger sequencing and next generation sequencing methods. Tyrosine kinase inhibitors erlotinib and gefitinib are associated with improved progression free survival over chemotherapy in EGFR L858R patients. NCCN guidelines recommend (category 1) erlotinib and gefitinib for NSCLC with sensitizing EGFR mutations, along with afatinib and osimertinib.
Explanation if clinical significance is other or drug response	Supports Sensitivity/Response; Drugs: Erlotinib and Gefitinib
Condition comment	-
Clinical significance	Drug Response
Collection method	literature only
Allele origin	somatic
Affected status	unknown
Comment	Tier I Level A
Private comment	-
ClinVarAccession	TBD
