# Supplementary figures and images for: Adapting crowdsourced clinical cancer curation in CIViC to the ClinGen minimum variant level data community‐driven standards
Source: Hum Mutat. 2018 Oct 11;39(11):1721–32. doi: 10.1002/humu.23651 (PMC6282863; doi:10.1002/humu.23651)

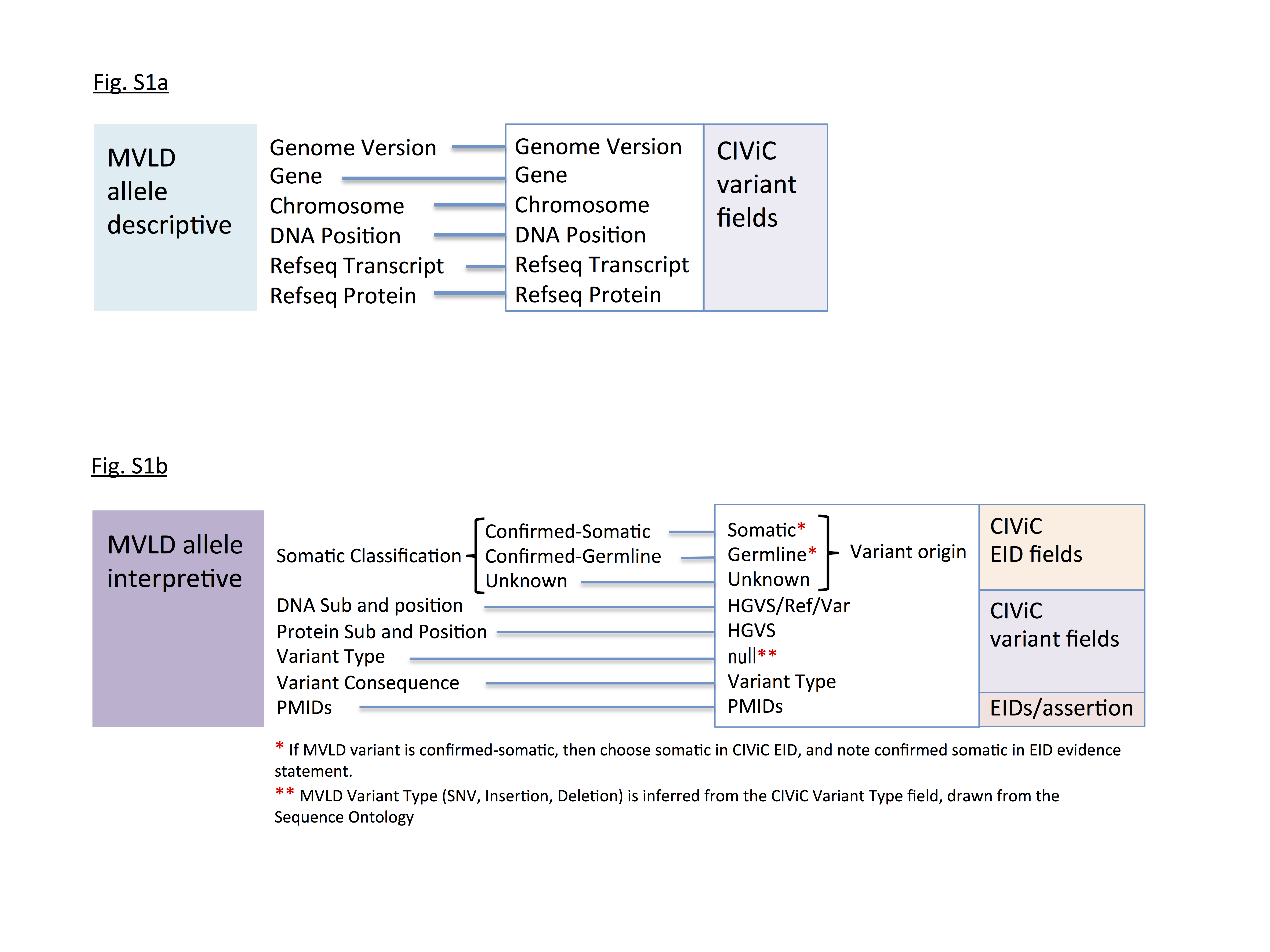

Supplement: Supplementary file 2 — Figure S1. Field by field mapping of MVLD into CIViC. (a) MVLD Allele Descriptive fields map into existing CIViC fields with no alteration. (b) MVLD Allele Interpretive fields map into CIViC fields with some conditions on the mapping for Somatic Classification and Variant Type fields. (c) MVLD Somatic Interpretive fields map into CIViC fields with some conditions on the mapping for Biomarker Class fields and the exception of expert opinion Sub Level of Evidence, which has no analogue in the CIViC format. [file HUMU-39-1721-s001.tiff]

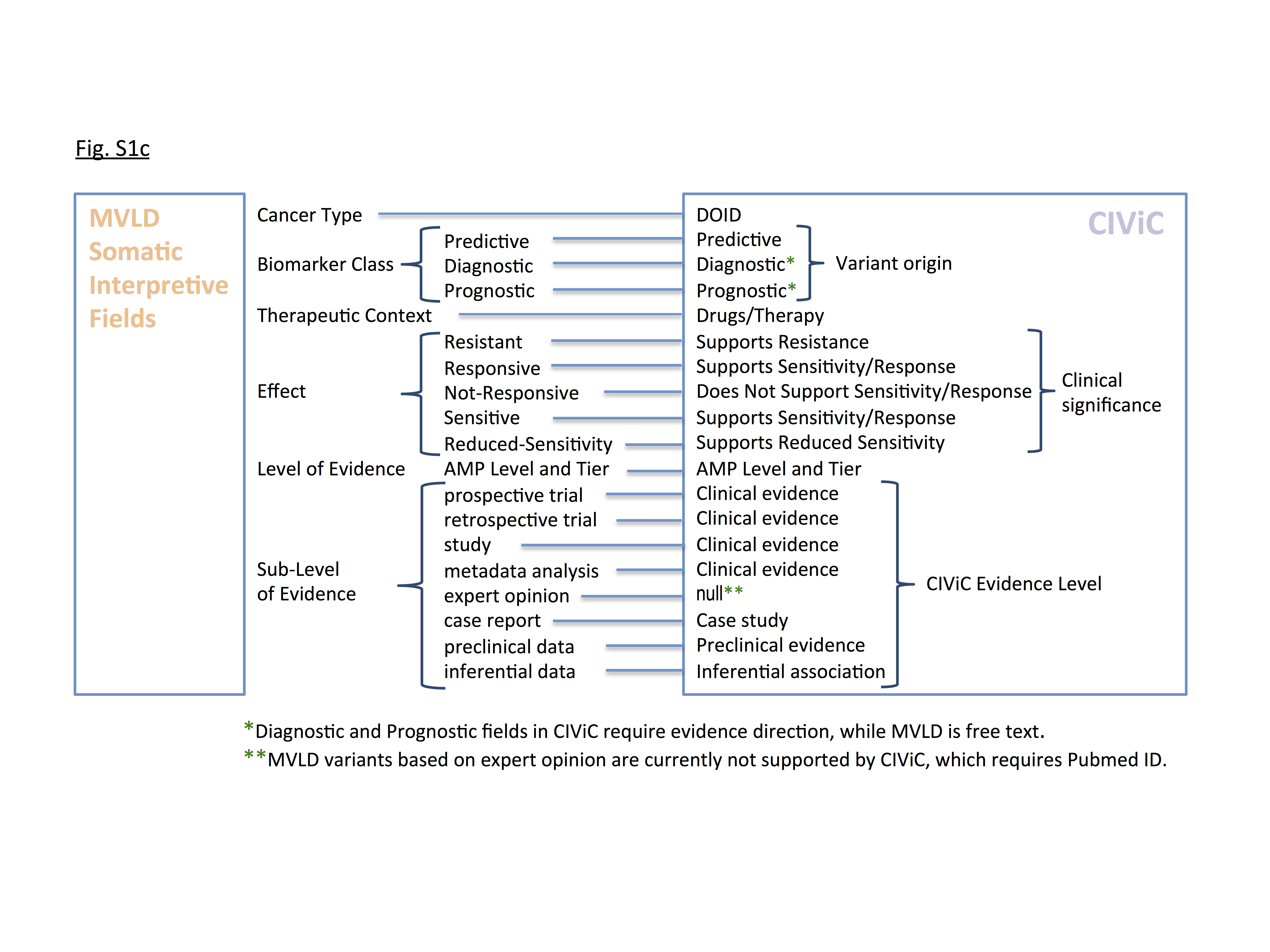

Supplement: Supplementary file 3 — Supporting Information [file HUMU-39-1721-s002.tiff]

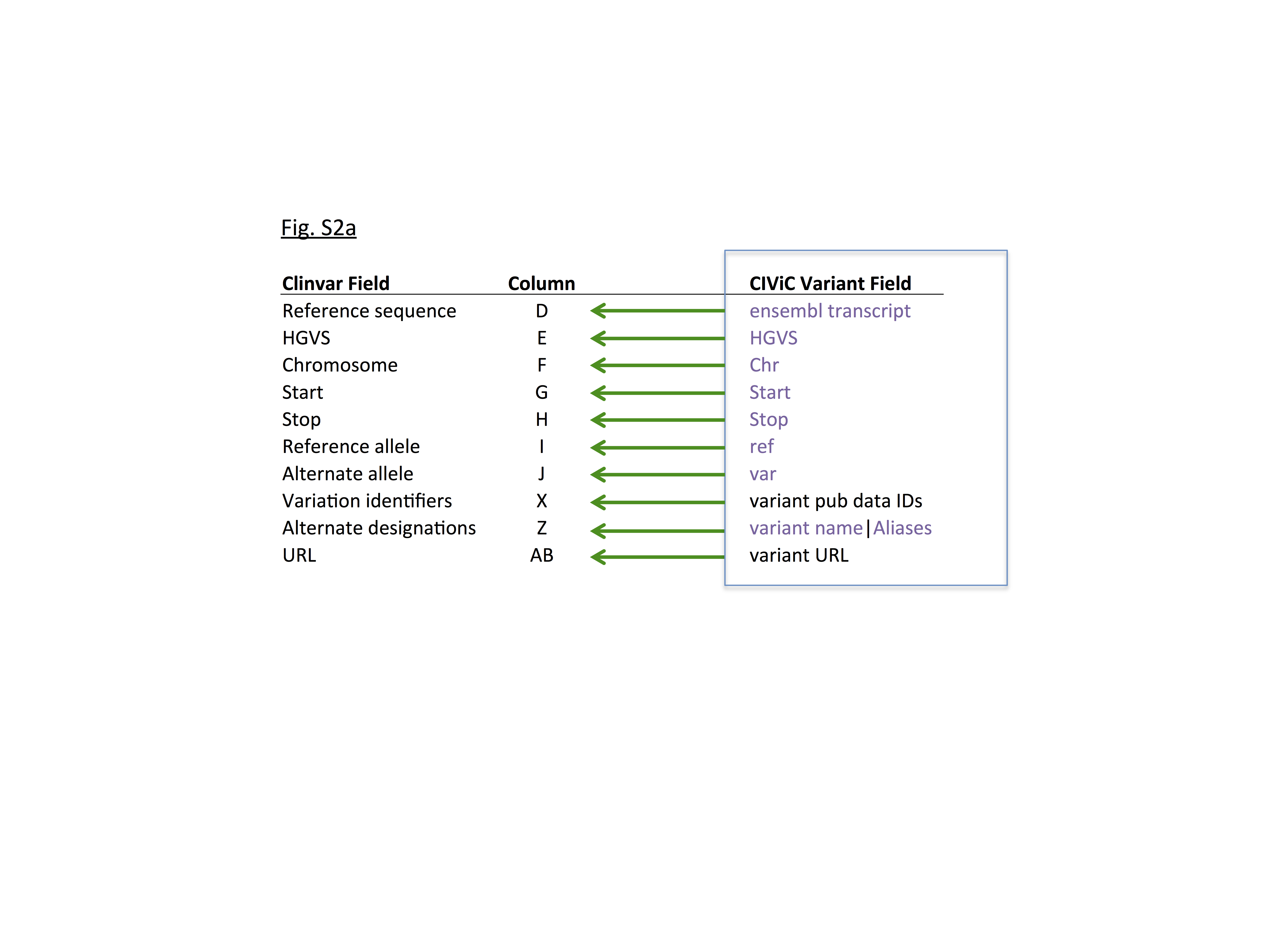

Supplement: Supplementary file 4 — Figure S2. Protocol for automated generation of ClinVar submission on a field by field basis. Three types of fields are distinguished by the process of generating ClinVar submissions from CIViC fields. None of the required ClinVar submission fields is impossible to generate using data either directly obtained or derived from CIViC assertion and related fields. (a) CIViC Variant fields map directly into a subset of required for ClinVar submission. (b) CIViC Assertion fields map into a subset of ClinVar submission fields non‐overlapping with the variant fields, and requires some logic to generate Condition ID Type and Condition ID Value fields. (c) A third type of ClinVar submission field has no direct analogue in CIViC fields but is filled using logic based on CIViC fields or free text. [file HUMU-39-1721-s003.tiff]

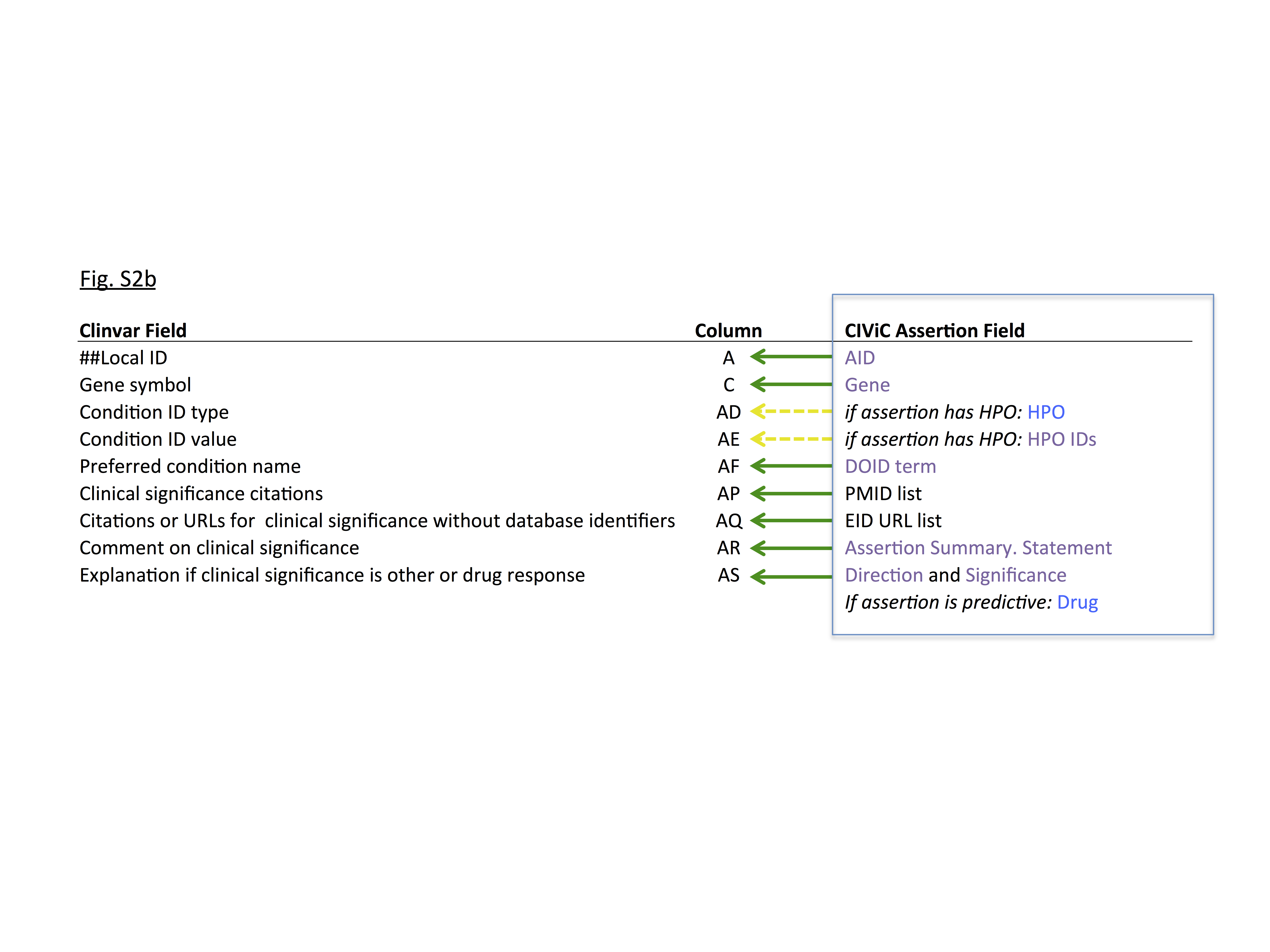

Supplement: Supplementary file 5 — Supporting Information [file HUMU-39-1721-s004.tiff]

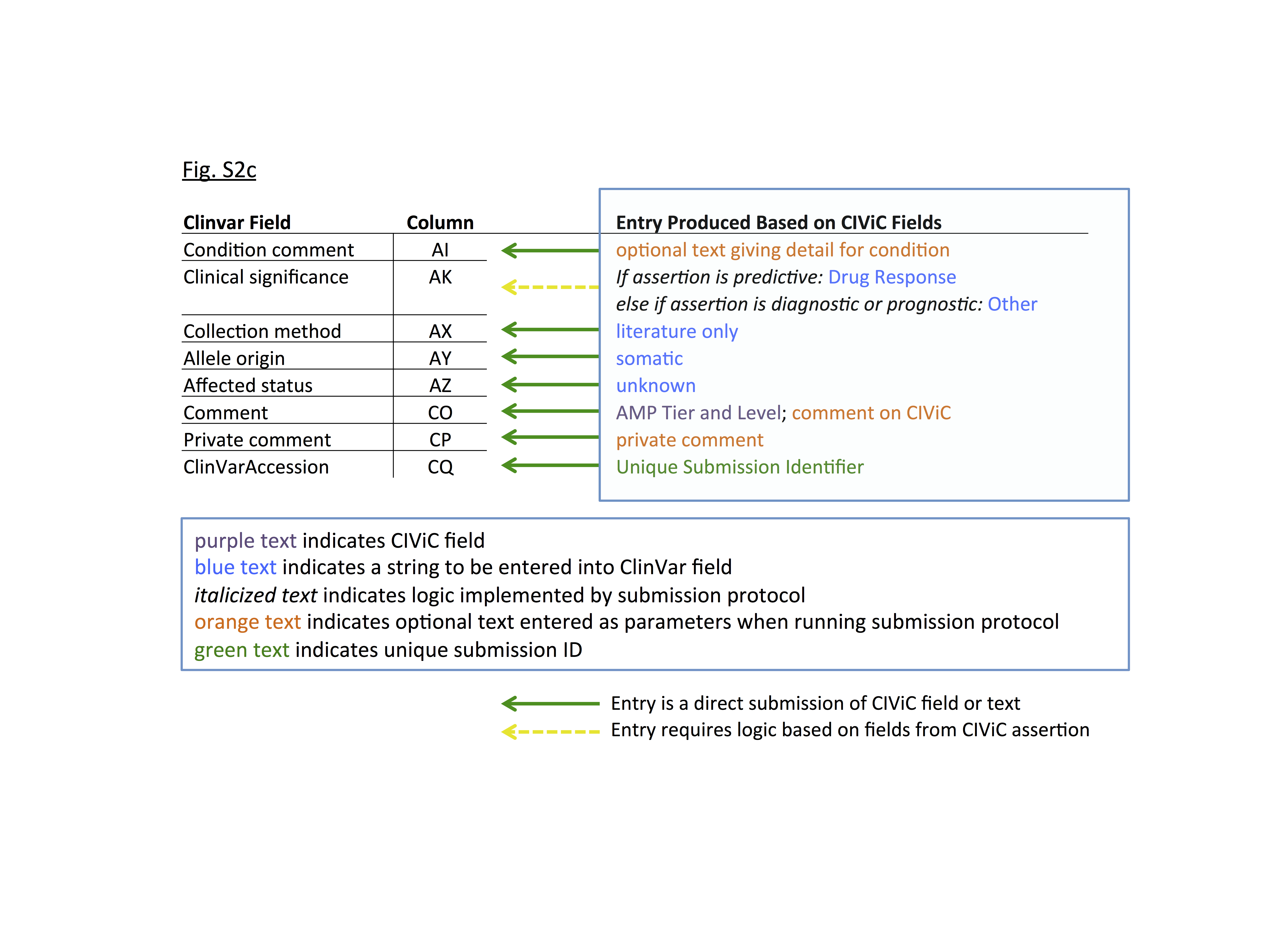

Supplement: Supplementary file 6 — Supporting Information [file HUMU-39-1721-s005.tiff]
