# Supplementary material for: Adapting crowdsourced clinical cancer curation in CIViC to the ClinGen minimum variant level data community‐driven standards
Source: Hum Mutat. 2018 Oct 11;39(11):1721–32. doi: 10.1002/humu.23651 (PMC6282863; doi:10.1002/humu.23651)
Supplement: Supplementary file 7 — Figure S3. CIViC fields transformed to ClinVar format. A sample transformation from fields of a CIViC assertion (AID5) following the protocol for automated ClinVar submission in the civic2clinvar python tool. [file HUMU-39-1721-s006.pdf]

Figure S3

ClinVar Field	CIViC data example from AID5
Reference sequence	NM_005228.4
HGVS	NC_000007.13:g.55259515T>G,NM_005228.4:c.2573T>G,ENST00000275493.2:c.2573T>G,NP_005219.2:p.Leu858Arg
Chromosome	7
Start	55259515
Stop	55259515
Reference allele	T
Alternate allele	G
Variation identifiers	dbSNP:rs121434568; COSMIC:COSM6224; ClinVar:16609
Alternate designations	LEU858ARG; RS121434568
URL	https://civicdb.org/events/genes/19/summary/variants/33/summary
##Local ID	AID5
Gene symbol	EGFR
Condition ID type	-
Condition ID value	-
Preferred condition name	non-small cell lung carcinoma (DOID:3908)
Clinical significance citations	PMID:24868098;PMID:18509184;PMID:20038723;PMID:24893891;PMID:15329413;PMID:19147750;PMID:22370314;PMID:24736073;PMID:27102076;PMID:27032107;PMID:21132006;PMID:17877814;PMID:22285168;PMID:24868098
Citations or URLs for clinical significance without database identifiers	https://civicdb.org/links/evidence/276|https://civicdb.org/links/evidence/1665|https://civicdb.org/links/evidence/2621|https://civicdb.org/links/evidence/2625|https://civicdb.org/links/evidence/2624|https://civicdb.org/links/evidence/3811|https://civicdb.org/links/evidence/2634|https://civicdb.org/links/evidence/229|https://civicdb.org/links/evidence/4291|https://civicdb.org/links/evidence/4290|https://civicdb.org/links/evidence/4285|https://civicdb.org/links/evidence/4265|https://civicdb.org/links/evidence/885|https://civicdb.org/links/evidence/2994
Comment on clinical significance	Non-small cell lung cancer with EGFR L858R mutation is sensitive to erlotininb or gefitinib. L858R is among the most common sensitizing EGFR mutations in NSCLC, and is assessed via DNA mutational analysis including Sanger sequencing and next generation sequencing methods. Tyrosine kinase inhibitors erlotinib and gefitinib are associated with improved progression free survival over chemotherapy in EGFR L858R patients. NCCN guidelines recommend (category 1) erlotinib and gefitinib for NSCLC with sensitizing EGFR mutations, along with afatinib and osimertinib.
Explanation if clinical significance is other or drug response	Supports Sensitivity/Response; Drugs: Erlotinib and Gefitinib
Condition comment	-
Clinical significance	Drug Response
Collection method	literature only
Allele origin	somatic
Affected status	unknown
Comment	Tier I Level A
Private comment	-
ClinVarAccession	TBD
